# Current News

**Published:** 1898-10

**Authors:** 


### Current News.

### NATIONAL ASSOCIATION OF DENTAL EXAMINERS.

Notice is hereby given that the next annual meeting of the National Association of Dental Examiners will be held at Washington, D. C., commencing at ten o'clock A.M., Thursday, October 13, and continuing in session the 14th and 15th. The head-quarters will be at "The Hamilton," Fourteenth and K Streets, opposite Franklin Park. The rates will be two dollars and two dollars and fifty cents per day.

Members can communicate with Dr. H. B. Noble for additional information regarding accommodations.

The poll-vote closed August 9, with seventy-two votes for Washington, twenty for Louisville, seventeen for Chicago, and twelve for Omaha, balance scattering.

Charles A. Meeker, D.D.S.,
*Secretary.*

29 Fulton Street, Newark, N. J.

---

### NORTHEASTERN DENTAL ASSOCIATION.

The Fourth Annual Meeting of the Northeastern Dental Association will be held in Hartford, Conn., October 19 and 20, 1898. The Executive Committee have arranged for a programme of papers and clinics; also extensive plans have been made to have a good exhibit of dental goods and medicines. A large gathering is desired and confidently looked for.

Edgar O. Kinsman,

*Secretary.*

Cambridge, Mass.

